# Depressive symptoms and quality of life in Multimorbidity: A Scoping Review of Community-Based Interventions

**DOI:** 10.1093/geroni/igaf122.4095

**Published:** 2025-12-31

**Authors:** Benita Bamgbade, Irina Mindlis, Atami De Main, Hanzhang Xu, Wenhui Zhang, Lindsey Randall, Abigail Sallings, Isabella Thoene

**Affiliations:** Northeastern University, Boston, Massachusetts, United States; Rutgers University, New York, New York, United States; Weill Cornell Medicine, New York, New York, United States; Duke University, Durham, North Carolina, United States; Emory University, Atlanta, Georgia, United States; Mayo Clinic, Rochester, Minnesota, United States; University of Florida, Gainesville, Florida, United States; University of Florida, Gainesville, Florida, United States

## Abstract

Depressive symptoms and poor quality of life (QoL) are common among over 65% of older adults living with multimorbidity. Community-based interventions have the potential for scalability and may be more accessible for older adults with complex needs compared to interventions delivered in healthcare settings. This scoping review aims to describe the literature on community-based interventions targeting depressive symptoms and QoL for adults with multimorbidity. Six databases were searched for studies published in 2015 or later, focusing on interventions in community-based settings (e.g., community-based organizations, senior centers, retirement communities). Studies focusing on a single condition or an index condition with comorbidities were excluded. Of 7,829 screened studies, 173 articles were included in full-text review. About half of the studies were conducted in high-income English-speaking countries and 11% in low and middle-income countries (LMIC). Study samples consisted mostly of older adults with varied multimorbidity profiles that included diabetes, cardiovascular disease, mental health disorders, and frailty. Nearly 40% of studies reported physical activity and patient education interventions, while peer-led/community health worker interventions were the least reported intervention. This review highlights a gap in studies conducted in LMIC where the burden of multimorbidity is high and resources are limited, and a gap in interventions that are community member-led. More research is needed to understand how these scalable, cost-effective community-based interventions may address the needs of multimorbidity patients in LMIC and how leveraging community members may strengthen interventions in addressing depressive symptoms and QoL in patients with multimorbidity.

